# Measuring change in mathematics enjoyment: longitudinal invariance, local dependence of negatively worded items, and complex-sample inference

**DOI:** 10.3389/fpsyg.2026.1971121

**Published:** 2026-10-26

**Authors:** Georgios Sideridis, Mohammed Alghamdi

**Affiliations:** 1 Boston Children’s Hospital, BARD, Harvard Medical School, Boston, MA, United States; 2 Department of Self-Development Skills, King Saud University, Riyadh, Saudi Arabia

**Keywords:** jackknife repeated replication, local dependence, longitudinal measurement invariance, mathematics enjoyment, non-cognitive constructs, ordinal confirmatory factor analysis, TIMSS

## Abstract

**Introduction:**

Inferences about developmental change require comparable measurement across occasions. We examined one-year change in mathematics enjoyment from Grade 4 to Grade 5 in the TIMSS 2023 Longitudinal Study.

**Methods:**

Linked files comprised 44,325 students from nine education systems; 43,979 had a positive sampling weight and at least one focal response. Seven positively worded item contents were common to 2023 and 2024, whereas two negatively worded items appeared only in 2023. Within each system, ordinal longitudinal confirmatory factor models incorporated sampling weights, correlated residuals for matching contents, and configural through scalar invariance testing. Separately, design-based uncertainty for latent change was estimated by refitting the scalar model across all 1,604 replicates from 802 paired jackknife zones.

**Results:**

Weighted WLSMV diagnostics supported scalar invariance in every system (scalar CFI = 0.994–0.997; RMSEA = 0.023–0.053; SRMR = 0.019–0.034; maximum absolute change in CFI across successive steps = 0.00050). A baseline one-factor model combining all nine items fit markedly worse; allowing residual dependence between the two negatively worded items yielded standardized residual correlations of 0.413–0.715. Within-system latent change was negative in all nine systems (−0.553 to −0.117 system-specific Grade 4 SD), with all paired-JRR 95% confidence intervals excluding zero. Estimates retained their sign under every response-completeness and weighting definition. Plausible-value-pooled response models produced the same substantive selection conclusion as a sensitivity model using the mean of the five achievement plausible values.

**Discussion:**

The content-matched seven-item core supports within-system longitudinal comparison; no cross-country equality of change magnitudes was tested. Local dependence between the negatively worded items may reflect wording direction, shared aversive content, or both.

## 1 Introduction

Enjoyment of learning mathematics is an activity-related achievement emotion and a constituent of intrinsic task value. Control–value theory specifies that enjoyment arises when a student appraises an achievement activity as sufficiently controllable and as subjectively valuable (Pekrun, 2006). Expectancy–value accounts assign intrinsic value an analogous role in organizing choice, persistence, and engagement (Wigfield et al., 1997; Eccles and Wigfield, 2020). Enjoyment covaries with adaptive engagement and with achievement, and longitudinal evidence indicates that control and value appraisals predict subsequent enjoyment and boredom in mathematics (Putwain et al., 2018). Mathematics enjoyment is therefore treated here as a substantive non-cognitive outcome rather than as an ancillary correlate of achievement.

Developmental research has repeatedly documented decrements in academic intrinsic motivation and in learning enjoyment across the school years. Longitudinal studies have reported declining mathematics intrinsic motivation from childhood through adolescence (Gottfried et al., 2007), decreasing task values across the elementary grades (Wigfield et al., 1997), and broad reductions in learning enjoyment and learning effort across primary and secondary education (Hawrot et al., 2026). An observed decrement in a scale score is interpretable as construct change, however, only under the assumption that the instrument functions equivalently on the occasions being compared. If item thresholds, loadings, or response processes covary with age, an apparent mean trajectory confounds substantive development with differential measurement.

Longitudinal factorial invariance provides the formal apparatus for separating these two sources. Configural invariance establishes a common measurement pattern; metric invariance constrains the indicator–construct relations; scalar invariance additionally aligns indicator locations and thereby licenses the comparison of latent means across occasions (Widaman et al., 2010; Putnick and Bornstein, 2016). The requirement is particularly consequential for ordered categorical indicators. Identification constraints that are inconsequential within a single ordinal confirmatory factor analysis (CFA) can operate as unintended restrictions when a sequence of invariance models is derived from one baseline parameterization. Wu and Estabrook (2016) developed an identification framework in which each level of ordinal invariance is defined independently of arbitrary baseline constraints, and Liu et al. (2017) extended the corresponding testing sequence to repeated measures. This framework is appropriate for the four-category agreement format used in the TIMSS student questionnaire.

Item wording constitutes a second threat to developmental interpretation. Reverse-worded items are typically included to attenuate acquiescent responding, but reversing item direction can introduce comprehension demands, inattentive responding, or response styles that generate method-specific covariance. Positively and negatively keyed indicators frequently require method factors or correlated uniquenesses even when they were written to represent a single trait (Lindwall et al., 2012). Psychometric and experimental evidence further questions whether reversed items improve scale quality, particularly where negation alters the response process rather than simply inverting the construct direction (Suárez-Álvarez et al., 2018). Where the wording composition of a scale differs between occasions, the comparison of conventional composite scores is therefore not defensible.

The TIMSS 2023 Longitudinal Study permits these issues to be examined in a large, multinational sample of students assessed on two occasions. TIMSS reassessed the Grade 4 students sampled in 2023 one school year later, when they were generally enrolled in Grade 5 (Fishbein et al., 2025; Fishbein et al., 2026). Nine education systems participated: Georgia, Italy, Jordan, the Republic of Korea, Kosovo, Montenegro, North Macedonia, Slovenia, and Sweden. The 2023 Students Like Learning Mathematics item set comprised seven positively worded indicators and two negatively worded indicators. The 2024 longitudinal questionnaire retained the seven positive contents but not the two negative contents. A content-matched seven-item core can therefore be examined longitudinally, while the baseline data permit a diagnostic of local dependence between the two negatively worded items. Because wording direction and their shared avoidance/boredom content are confounded, the baseline comparison cannot isolate a wording-method effect.

Large-scale longitudinal data also require the sampling design to be carried into uncertainty estimation. TIMSS employs stratified multistage samples in which students are clustered within schools (Siegel and Foy, 2024) and supplies a jackknife zone and replicate indicator for every student. Within a zone, a replicate weight vector doubles the weight of one school of a paired set and assigns zero weight to the other; sampling variance is obtained from squared deviations of replicate estimates from the full-sample estimate (Fishbein et al., 2024). We therefore re-estimated the selected scalar model under every prescribed replicate so that the latent-change estimator—not only a manifest mean—was propagated through the design-based variance calculation. This inference step is distinct from evaluating measurement-model fit.

The present study examined the measurement and one-year development of mathematics enjoyment. Four research questions were addressed. First, does the seven-item positive core exhibit configural, threshold, metric, and scalar invariance from Grade 4 in 2023 to Grade 5 in 2024 within each education system? Second, do the two negatively worded 2023 items exhibit residual dependence after conditioning on a common enjoyment factor? Third, conditional on scalar invariance, how does latent mathematics enjoyment change over one school year within each system once TIMSS sampling uncertainty is incorporated? Fourth, are conclusions robust to item-response completeness, sampling weights, and observed follow-up selection? We expected the positive core to support at least metric invariance, treated scalar invariance as an empirical question, and expected the two negatively worded items to display local dependence. The nine-system design was used to examine replication of the direction of within-system change; it was not used to test equality of change magnitudes across systems.

## 2 Materials and methods

### 2.1 Data source and longitudinal linkage

The public-use SPSS student databases of the TIMSS 2023 Longitudinal Grade 4 and 5 release were analyzed (IEA International Association for the Evaluation of Educational Achievement and TIMSS and PIRLS International Study Center at Boston College, 2026). The study constituted an optional extension of TIMSS 2023. National centers tracked the students sampled in Grade 4 in 2023 and reassessed the same students in Grade 5 in 2024, including students who had changed class or school and could be located (Fishbein et al., 2025). Students retained the longitudinal identifier IDSTUD across occasions. An audit of the country files returned one record per identifier and no duplicate identifiers.

The release comprised Georgia, Italy, Jordan, Kosovo, Montenegro, North Macedonia, the Republic of Korea, Slovenia, and Sweden, totaling 44,325 linked records across nine education-system panels. The analytic sample for the confirmatory factor models retained records with a positive student sampling weight and at least one observed focal item across the two occasions, which yielded 43,979 students. System-specific item-response counts are reported in Table 1. Retention of the same identifier in the linked database does not imply that every focal item was observed on both occasions, and this distinction was preserved throughout the analysis.

**Table 1:** Longitudinal linkage and focal-response flow by education system.

Education system	Linked *N*	Analytic *N*	Baseline only	Follow-up only	Both waves	Neither	Pairwise *N*
Georgia	4,491	4,451	651	237	3,563	40	3,242–3,981
Italy	4,610	4,578	320	351	3,907	32	3,819–4,230
Jordan	6,172	6,120	976	280	4,864	52	4,236–5,390
Kosovo	4,747	4,708	471	246	3,991	39	3,562–4,192
Montenegro	4,559	4,517	351	279	3,887	42	3,490–3,997
North Macedonia	4,866	4,816	535	345	3,936	50	3,332–3,999
Republic of Korea	4,507	4,500	806	159	3,535	7	3,489–4,322
Slovenia	5,015	4,975	635	374	3,966	40	3,861–4,537
Sweden	5,358	5,314	678	361	4,275	44	4,180–4,908
Total	44,325	43,979	5,423	2,632	35,924	346	3,242–5,390

Counts are among records with a positive student sampling weight. Analytic N includes at least one focal response at either wave. Pairwise *N* is the minimum–maximum number contributing to any pair of the 14 longitudinal indicators.

TIMSS reported generally low weighted attrition in the longitudinal study, although follow-up loss was higher in Jordan than in most other participating systems (Fishbein et al., 2025). Because the outcome of the present analysis was a questionnaire construct rather than an imputed achievement estimate, observed questionnaire responses were analyzed directly and response selection was evaluated in separate diagnostic models (Section 2.7).

### 2.2 Measures

The longitudinal construct was indicated by seven item contents administered on both occasions: enjoying learning mathematics, learning interesting things in mathematics, liking mathematics, liking schoolwork that involves numbers, liking mathematics problems, looking forward to mathematics lessons, and mathematics being a favorite subject. The 2023 Grade 4 variables were ASBM02A and ASBM02D–ASBM02I; the corresponding 2024 Grade 5 variables were ASBLM02A–ASBLM02G (Supplementary Table S10). Responses were recorded on four ordered agreement categories and were recoded so that 1 denoted the least enjoyment-consistent response and 4 the most enjoyment-consistent response. Throughout this article, mathematics enjoyment is used as a concise label for this positive-item core. Because the contents also refer to interest, liking, and favorite-subject status, the construct should be interpreted as students’ positive affective valuation of mathematics rather than as a pure momentary achievement emotion.

The 2023 item set additionally contained two negatively worded contents, “wish [I] did not have to study mathematics” and “mathematics is boring” (ASBM02B and ASBM02C), which were keyed so that higher values denoted greater enjoyment. These contents were excluded from the longitudinal core because no corresponding items were administered in the 2024 longitudinal questionnaire; they were retained for the baseline local-dependence diagnostic. The TIMSS 2023 Longitudinal Technical Report describes a committee-led revision of the 2024 questionnaires and general revision goals, but explicitly notes that its summary does not provide a list of item-by-item edits (Fishbein et al., 2025, p. 5). We found no published TIMSS rationale specific to omission of ASBM02B and ASBM02C and therefore treat their absence as a documented design difference, not as evidence that TIMSS removed them for psychometric reasons.

### 2.3 Longitudinal measurement model

All models were estimated separately within each education system. This specification aligned the measurement comparison with the repeated observations of students nested within a system and avoided the assumption of cross-national scalar invariance, which the design was not intended to evaluate. Each longitudinal model specified two correlated latent enjoyment factors, one for Grade 4 in 2023 and one for Grade 5 in 2024, with the seven contemporaneous indicators loading on the factor of their own occasion.

The seven indicators were treated as ordered categorical variables generated by underlying continuous latent response variables. For student *i*, item content *j* (*j* = 1, …, 7), and occasion *t* (*t* = 1, 2), the latent response variable was specified as shown in Equation (1):

$$y_{\mathrm{ijt}}^{*}=\nu_{\mathrm{jt}}+\lambda_{\mathrm{jt}}\;\eta_{\mathrm{it}}+\varepsilon_{\mathrm{ijt}},$$ (1)

where $\nu_{\mathrm{jt}}$ denotes the latent-response intercept, $\lambda_{\mathrm{jt}}$ the factor loading, $\eta_{\mathrm{it}}$ the latent enjoyment score, and $\varepsilon_{\mathrm{ijt}}$ the unique component. The observed four-category response was related to the latent response variable through a set of three free thresholds, as shown in Equation (2):

$$y_{\mathrm{ijt}}=c\Leftrightarrow \tau_{\mathrm{jt}}^{\left(c-1\right)}<y_{\mathrm{ijt}}^{*}\leq \tau_{\mathrm{jt}}^{\left(c\right)},\;\;\;\;c=1,\ldots ,4,$$ (2)

with $\tau_{\mathrm{jt}}^{\left(0\right)}=-\infty$ and $\tau_{\mathrm{jt}}^{\left(4\right)}=+\infty$ . Indicator-specific stability not accounted for by the common factors was represented by freely estimated residual covariances between matching item contents across occasions, whereas residual covariances between non-matching contents were fixed at zero, as shown in Equation (3):

$$\mathrm{cov}\;(\varepsilon_{\mathrm{ij}1},\varepsilon_{\mathrm{ij}\prime 2})=\{\begin{array}{ll} \theta_{j}, & j=j^{\prime },\\ 0, & j\neq j^{\prime }. \end{array}$$ (3)

Omission of these parameters would attribute occasion-specific indicator variance to the common factors and would bias the estimated stability of the construct.

The latent factors were assumed to follow a bivariate distribution with mean vector $\alpha ={\left(\alpha_{1},\alpha_{2}\right)}^{⊤}$ and covariance matrix Ψ containing the variances $\psi_{11}$ and $\psi_{22}$ and the covariance $\psi_{21}$ . The metric of the construct was set at the first occasion by fixing $\alpha_{1}=0$ and $\psi_{11}=1$ , so that the estimand of substantive interest,

$$\Delta =\alpha_{2}-\alpha_{1}=\alpha_{2},$$ (4)

expressed the one-year change in latent enjoyment in Grade 4 latent standard deviation units, as defined in Equation (4):

Models were estimated by diagonally weighted least squares with mean and variance-adjusted test statistics (WLSMV) under the theta parameterization. The student sampling weight (TOTWGT) was normalized to a mean of one within each panel. TIMSS describes TOTWGT as the overall student sampling weight and its weighting components as incorporating school, class-, and student-level participation adjustments (Fishbein et al., 2026; Siegel and Foy, 2024). It does not impute or otherwise correct missing responses to the focal questionnaire items, and it is not a separate adjustment for 2024 focal-item attrition. Missing ordinal responses were therefore handled with pairwise-present information in WLSMV and examined in separate response restrictions and selection models. Every model was required to converge and satisfy the post-estimation admissibility check.

### 2.4 Invariance testing and model evaluation

Longitudinal invariance was evaluated through the following nested sequence: (a) a configural model, in which thresholds and loadings were freely estimated at each occasion; (b) a threshold-invariance model, in which corresponding thresholds were constrained to equality across occasions, $\tau_{j1}^{\left(c\right)}=\tau_{j2}^{\left(c\right)}$ ; (c) a metric model, which additionally imposed $\lambda_{j1}=\lambda_{j2}$ ; and (d) a scalar model, which additionally imposed $\nu_{j1}=\nu_{j2}$ . At each level the identification constraints on the latent-response intercepts, unique variances, and latent moments were adjusted according to Wu and Estabrook (2016), as extended to repeated measures by Liu et al. (2017), so that successive models were properly nested and the Grade 5 latent mean became estimable only once thresholds and loadings had been established as invariant. This identification strategy avoids the arbitrary baseline restrictions that arise when an entire invariance sequence is derived from a single reference parameterization.

Model fit was summarized by scaled chi-square, CFI, TLI, RMSEA with its 90% confidence interval, and SRMR. Table 2 reports the complete configural-to-scalar sequence and successive changes for every system. Invariance decisions emphasized changes across approximate-fit indices because chi-square differences are highly sensitive in these samples. Chen’s (2007) reference values were treated as diagnostics, not universal rules. These fit statistics incorporate sampling weights but do not include a separate school-cluster correction to their reference distributions. Accordingly, the invariance conclusion is evidence from weighted WLSMV fit diagnostics; the paired-jackknife analysis described below provides design-based uncertainty for latent change conditional on the selected scalar model and is not a design-adjusted model-selection test.

**Table 2:** Complete weighted WLSMV longitudinal invariance sequence by education system.

System	Model	*χ*^2^ (df)	CFI	TLI	RMSEA [90% CI]	SRMR	ΔCFI	ΔRMSEA	ΔSRMR
Georgia	Configural	372.2 (69)	0.9954	0.9939	0.031 [0.028, 0.035]	0.0324	—	—	—
Georgia	Thresholds	390.7 (76)	0.9952	0.9943	0.031 [0.028, 0.034]	0.0324	−0.00017	−0.00092	0.00000
Georgia	Metric	400.6 (82)	0.9951	0.9946	0.030 [0.027, 0.032]	0.0323	−0.00006	−0.00096	−0.00010
Georgia	Scalar	403.9 (88)	0.9952	0.9950	0.028 [0.026, 0.031]	0.0323	0.00004	−0.00115	0.00005
Italy	Configural	701.5 (69)	0.9964	0.9952	0.045 [0.042, 0.048]	0.0229	—	—	—
Italy	Thresholds	728.0 (76)	0.9963	0.9956	0.043 [0.040, 0.046]	0.0229	−0.00011	−0.00146	0.00000
Italy	Metric	742.7 (82)	0.9962	0.9958	0.042 [0.039, 0.045]	0.0229	−0.00005	−0.00134	0.00001
Italy	Scalar	814.2 (88)	0.9959	0.9957	0.042 [0.040, 0.045]	0.0231	−0.00037	0.00050	0.00019
Jordan	Configural	334.2 (69)	0.9965	0.9954	0.025 [0.022, 0.028]	0.0242	—	—	—
Jordan	Thresholds	350.1 (76)	0.9964	0.9957	0.024 [0.022, 0.027]	0.0242	−0.00012	−0.00078	0.00000
Jordan	Metric	356.0 (82)	0.9964	0.9960	0.023 [0.021, 0.026]	0.0242	0.00000	−0.00091	−0.00001
Jordan	Scalar	379.7 (88)	0.9962	0.9961	0.023 [0.021, 0.026]	0.0239	−0.00023	−0.00009	−0.00022
Kosovo	Configural	401.7 (69)	0.9943	0.9925	0.032 [0.029, 0.035]	0.0337	—	—	—
Kosovo	Thresholds	437.7 (76)	0.9938	0.9926	0.032 [0.029, 0.035]	0.0337	−0.00050	−0.00020	0.00000
Kosovo	Metric	445.7 (82)	0.9937	0.9931	0.031 [0.028, 0.034]	0.0337	−0.00003	−0.00110	0.00002
Kosovo	Scalar	427.7 (88)	0.9942	0.9940	0.029 [0.026, 0.031]	0.0336	0.00041	−0.00206	−0.00009
Montenegro	Configural	612.6 (69)	0.9965	0.9953	0.042 [0.039, 0.045]	0.0220	—	—	—
Montenegro	Thresholds	667.4 (76)	0.9962	0.9954	0.042 [0.039, 0.044]	0.0220	−0.00031	−0.00026	0.00000
Montenegro	Metric	701.3 (82)	0.9960	0.9955	0.041 [0.038, 0.044]	0.0220	−0.00018	−0.00062	−0.00001
Montenegro	Scalar	713.0 (88)	0.9959	0.9958	0.040 [0.037, 0.042]	0.0222	−0.00004	−0.00124	0.00018
North Macedonia	Configural	369.0 (69)	0.9971	0.9961	0.030 [0.027, 0.033]	0.0224	—	—	—
North Macedonia	Thresholds	382.7 (76)	0.9970	0.9964	0.029 [0.026, 0.032]	0.0224	−0.00007	−0.00110	0.00000
North Macedonia	Metric	411.4 (82)	0.9968	0.9964	0.029 [0.026, 0.032]	0.0223	−0.00022	−0.00007	−0.00004
North Macedonia	Scalar	408.0 (88)	0.9969	0.9967	0.027 [0.025, 0.030]	0.0222	0.00009	−0.00140	−0.00014
Republic of Korea	Configural	876.2 (69)	0.9966	0.9955	0.051 [0.048, 0.054]	0.0184	—	—	—
Republic of Korea	Thresholds	911.8 (76)	0.9965	0.9958	0.049 [0.047, 0.052]	0.0184	−0.00012	−0.00155	0.00000
Republic of Korea	Metric	982.9 (82)	0.9962	0.9958	0.049 [0.047, 0.052]	0.0185	−0.00028	−0.00002	0.00008
Republic of Korea	Scalar	1010.3 (88)	0.9961	0.9960	0.048 [0.046, 0.051]	0.0185	−0.00009	−0.00115	0.00003
Slovenia	Configural	1146.5 (69)	0.9949	0.9933	0.056 [0.053, 0.059]	0.0229	—	—	—
Slovenia	Thresholds	1180.0 (76)	0.9948	0.9937	0.054 [0.051, 0.057]	0.0229	−0.00013	−0.00199	0.00000
Slovenia	Metric	1201.6 (82)	0.9947	0.9941	0.052 [0.050, 0.055]	0.0229	−0.00007	−0.00165	−0.00001
Slovenia	Scalar	1310.2 (88)	0.9942	0.9940	0.053 [0.050, 0.055]	0.0230	−0.00049	0.00045	0.00008
Sweden	Configural	883.8 (69)	0.9963	0.9951	0.047 [0.044, 0.050]	0.0206	—	—	—
Sweden	Thresholds	906.9 (76)	0.9962	0.9955	0.045 [0.043, 0.048]	0.0206	−0.00007	−0.00178	0.00000
Sweden	Metric	954.9 (82)	0.9960	0.9956	0.045 [0.042, 0.047]	0.0207	−0.00019	−0.00060	0.00009
Sweden	Scalar	993.9 (88)	0.9959	0.9957	0.044 [0.042, 0.047]	0.0207	−0.00015	−0.00074	0.00000

*χ*^2^ is the scaled statistic. Δ values compare each model with the immediately preceding step; dashes indicate the configural model. Fit diagnostics incorporate normalized student weights but no separate school-cluster correction. Scaled WLSMV *χ*^2^ statistics need not increase monotonically across nested models because model-specific scaling corrections differ; the metric-to-scalar decreases for North Macedonia and Kosovo do not by themselves indicate a nesting error.

### 2.5 Baseline local-dependence diagnostics

Three WLSMV models were fitted to the 2023 Grade 4 items within each system: (a) a one-factor model for the seven positively worded items; (b) a one-factor model for all nine keyed items; and (c) the nine-item model with a residual covariance between the two negatively worded items. The final parameter represents their shared variance after conditioning on the common enjoyment factor. We compared scaled chi-square and degrees of freedom, CFI, TLI, RMSEA with its 90% confidence interval, SRMR, and residual-correlation discrepancies. This diagnostic establishes local dependence under the specified factor model; it cannot separate a response effect of negative wording from shared avoidance/boredom content. The standardized residual correlation was calculated using Equation (5):

$${\hat{\rho }}_{\varepsilon }=\frac{{\hat{\theta }}_{\mathrm{BC}}}{\sqrt{{\hat{\theta }}_{\mathrm{BB}}\;{\hat{\theta }}_{\mathrm{CC}}}},$$ (5)

where the numerator denotes the residual covariance between ASBM02B and ASBM02C and the denominator rescales it by their unique variances. The resulting standardized residual correlation quantifies local dependence. It does not identify whether that dependence is caused by negative wording, shared aversive content, or both.

### 2.6 Complex-sample variance estimation

Uncertainty in the latent change estimate was quantified by the paired jackknife repeated replication (JRR) procedure prescribed for TIMSS (Fishbein et al., 2024). Each student record carries a jackknife zone indicator $z_{i}\in \{1,\ldots ,H\}$ and a replicate indicator $r_{i}\in \{0,1\}$ that distinguishes the paired schools or pseudo-schools within a zone. Two replicate weight vectors were constructed for each zone *h*, as shown in Equation (6):

$$\begin{array}{l} w_{i}^{\left(h,1\right)}=\{\begin{array}{ll} 2w_{i}, & z_{i}=h,r_{i}=0,\\ 0, & z_{i}=h,r_{i}=1,\\ w_{i}, & z_{i}\neq h, \end{array}\;\text{and}\\ w_{i}^{\left(h,2\right)}=\{\begin{array}{ll} 0, & z_{i}=h,r_{i}=0,\\ 2w_{i}, & z_{i}=h,r_{i}=1,\\ w_{i}, & z_{i}\neq h. \end{array} \end{array}$$ (6)

Weights outside the focal zone were left unchanged. The scalar ordinal CFA was re-estimated in full under every replicate weight vector, so that the replicated statistic was the complete latent variable estimator rather than a manifest summary. Because WLSMV employs the diagonally weighted least squares objective for point estimation, the replicate runs used that objective while suppressing the model-based standard errors and global test statistics, which are redundant under replication. Denoting by $\hat{\Delta }$ the full-sample latent change estimate and by ${\hat{\Delta }}^{\left(h,m\right)}$ the estimate obtained under replicate *m* of zone *h*, the TIMSS sampling variance was computed as

$${\hat{\mathrm{Var}}}_{\mathrm{JRR}}\;(\hat{\Delta })=\frac{1}{2}\sum_{h=1}^{H}\sum_{m=1}^{2}{\left({\hat{\Delta }}^{\left(h,m\right)}-\hat{\Delta }\right)}^{2}.$$ (7)

Its square root was used as the sampling standard error, and 95% confidence intervals were formed as $\hat{\Delta }\pm 1.96\;{\hat{\mathrm{SE}}}_{\mathrm{JRR}}$ . We used a normal approximation for these intervals. Because both members of every zone were retained, the number of replicate models per system was twice the number of zones and ranged from 150 to 250. Equation 7 is the procedure documented for TIMSS 2023, with two replicates per zone and a factor of one half (Fishbein et al., 2024). The documented maximum of 125 zones permits 250 replicates, as in Montenegro. All 1,604 Grade 4-to-Grade 5 replicate models converged and satisfied the admissibility check, so no variance estimate was conditioned on the exclusion of failed replicates.

### 2.7 Sensitivity and response-selection analyses

The scalar model was re-estimated under four analytic definitions: (a) weighted, using all available focal responses; (b) weighted, requiring at least four of the seven items to be observed at each occasion; (c) weighted, requiring all seven items to be observed on both occasions; and (d) unweighted, using all available focal responses. The direction of latent change and the absolute displacement of each alternative estimate from the primary estimate were compared.

Observed follow-up selection was evaluated in two stages. A baseline enjoyment score was computed for students with at least four observed 2023 items, and follow-up response was defined as at least one observed 2024 focal item. Weighted baseline-score differences between responders and nonresponders were first standardized by the weighted baseline standard deviation. A design-based logistic response model, specified in Equation (8), was then fitted within each system using all paired JRR weights. Students missing any model covariate were excluded by complete-case analysis; final sample sizes are reported in Supplementary Table S8.

$$\text{logit}\;[\mathrm{Pr}\;(R_{i}=1)]=\gamma_{0}+\gamma_{1}E_{i}+x_{i}^{⊤}\gamma ,$$ (8)

Predictors were standardized baseline enjoyment, one mathematics achievement plausible value per fit, across all five plausible values, age, the TIMSS home-resources index, sex, frequency of speaking the test language at home, books in the home, student born in the country, and school absence. The model was fitted separately for each of the five mathematics plausible values while retaining JRR sampling variance within each fit. Coefficients and variances were combined with the TIMSS/Rubin multiple-imputation rule: the total variance equals mean within-plausible-value JRR variance plus (1 + 1/5) times the between-plausible-value variance (Rubin, 1987; Fishbein et al., 2026). As a sensitivity comparison, we also fitted the response model using each student’s mean of the five achievement plausible values, standardized within system, with the same covariates and paired-JRR variance estimation. This comparison does not propagate between-plausible-value uncertainty; the pooled models provide the primary response-selection results. These analyses characterize observed selection and do not establish an ignorable missingness mechanism.

### 2.8 Software

Analyses were conducted in R 4.5.3 (R Core Team, 2026), using lavaan 0.7–2 (Rosseel, 2012), semTools 0.5–9 (Jorgensen et al., 2026), and survey 4.5 (Lumley, 2004). The measEq.syntax function generated the invariance syntax under the Wu–Estabrook identification. Supplementary Data S1 contains item mapping and recoding, generated model syntax, replicate construction and scaling, convergence/admissibility checks, model outputs, and publication tables.

## 3 Results

### 3.1 Linked sample and observed item response

The nine files contained 44,325 linked records and 44,325 unique longitudinal identifiers, with no duplicates. Among positive-weight records, 7–52 students per system had no focal response at either wave, 320–976 contributed baseline-only responses, 159–374 contributed follow-up-only responses, and 3,535–4,864 contributed responses at both waves (Table 1; Supplementary Table S2). The primary models used 43,979 students with at least one focal response at either wave. Item response ranged from 81.7 to 96.3% in 2023 and from 78.5 to 92.2% in 2024 across system-item combinations (Supplementary Table S3). The ordinal correlation inputs were based on pairwise sample sizes ranging from 3,242 to 5,390 across systems (Supplementary Table S4). Thus, the linked cohort, the analytic sample, and the information supporting particular item pairs are distinct quantities.

### 3.2 Negatively worded items exhibited substantial local dependence

The seven-positive-item baseline models yielded CFI values of 0.992–0.997 and SRMR values of 0.017–0.030. Their RMSEA estimates were 0.036–0.093 (df = 14; 90% interval endpoints across systems = 0.029–0.099), indicating that absolute fit was not uniformly close by RMSEA. Low degrees of freedom can make RMSEA unstable, especially in small samples (Kenny et al., 2015), but the present samples were large; elevated RMSEA was therefore not dismissed. We evaluated CFI, TLI, SRMR, RMSEA intervals, and residual discrepancies jointly (Supplementary Table S5).

A single common factor fitted to all nine keyed items performed substantially worse (CFI = 0.903–0.988; RMSEA = 0.090–0.185; df = 27). Freeing one residual covariance between “wish [I] did not have to study mathematics” and “mathematics is boring” raised CFI to 0.992–0.997 and reduced RMSEA to 0.033–0.085 (df = 26). The negatively worded pair was the largest residual discrepancy in every unidimensional nine-item model. After the covariance was freed, the maximum absolute residual correlation was 0.049–0.088 and involved other item pairs (Supplementary Tables S5, S6). The comparative improvement is clear, but absolute RMSEA remained elevated in some augmented models, most notably Slovenia (RMSEA = 0.085, 90% CI [0.080, 0.090]).

The standardized residual correlation between the two negatively worded items was 0.413–0.715 (Table 3; Figure 1). This substantial dependence persisted after the common factor was modeled, so scoring the items in the enjoyment-consistent direction did not make them conditionally independent indicators. Because wording direction and aversive content were confounded, we refer to local dependence between the two items rather than attributing the association specifically to a reverse-wording method effect.

**Table 3:** Grade 4 baseline local-dependence diagnostic for the two negatively worded items.

System	*N*	*χ*^2^(df), 1F	CFI, 1F	RMSEA [90% CI], 1F	*χ*^2^(df), +resid	CFI, +resid	RMSEA [90% CI], +resid	SRMR, +resid	Residual r
Georgia	4,229	2299.8 (27)	0.931	0.141 [0.136, 0.146]	230.7 (26)	0.994	0.043 [0.038, 0.048]	0.025	0.684
Italy	4,231	1363.3 (27)	0.987	0.108 [0.103, 0.113]	547.3 (26)	0.995	0.069 [0.064, 0.074]	0.023	0.461
Jordan	5,877	5449.5 (27)	0.903	0.185 [0.181, 0.189]	204.3 (26)	0.997	0.034 [0.030, 0.039]	0.021	0.715
Kosovo	4,478	1015.9 (27)	0.947	0.090 [0.086, 0.095]	150.9 (26)	0.993	0.033 [0.028, 0.038]	0.030	0.556
Montenegro	4,254	3395.2 (27)	0.950	0.171 [0.166, 0.176]	447.5 (26)	0.994	0.062 [0.057, 0.067]	0.025	0.632
North Macedonia	4,513	3259.9 (27)	0.929	0.163 [0.158, 0.168]	291.3 (26)	0.994	0.048 [0.043, 0.053]	0.029	0.646
Republic of Korea	4,347	2137.4 (27)	0.982	0.134 [0.129, 0.139]	640.9 (26)	0.995	0.074 [0.069, 0.079]	0.018	0.527
Slovenia	4,608	2774.1 (27)	0.974	0.149 [0.144, 0.153]	888.2 (26)	0.992	0.085 [0.080, 0.090]	0.025	0.512
Sweden	4,958	1459.9 (27)	0.988	0.103 [0.099, 0.108]	783.8 (26)	0.994	0.077 [0.072, 0.081]	0.022	0.413

The augmented model differs only by a residual covariance between ASBM02B and ASBM02C. RMSEA entries include 90% confidence intervals. Residual r is the standardized covariance from the augmented model and may reflect wording direction, shared aversive content, or both.

**Figure 1:**
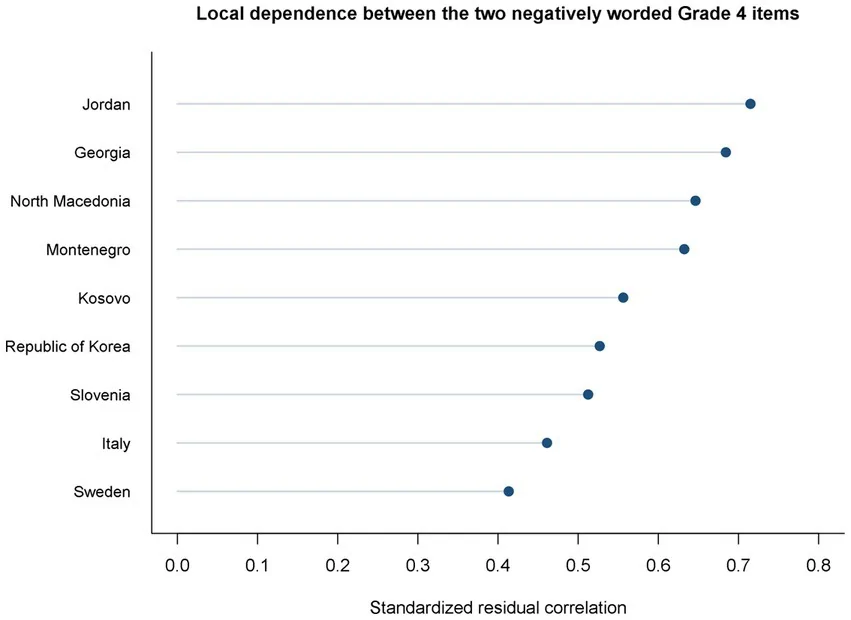
Local dependence between the two negatively worded Grade 4 baseline items. Points are standardized residual correlations between “wish [I] did not have to study mathematics” and “mathematics is boring” after conditioning on the common nine-item factor; wording direction and shared aversive content are not separable in this design.

### 3.3 The seven-item core supported scalar invariance in every panel

All 36 longitudinal models—four invariance levels in each of nine systems—converged and satisfied the post-estimation check. Table 2 and Supplementary Table S1 report the complete sequence. Scalar-model CFI was 0.994–0.997, RMSEA 0.023–0.053, and SRMR 0.019–0.034. Across all successive steps, the maximum absolute ΔCFI was 0.00050, the largest RMSEA increase was 0.00050, and the maximum absolute ΔSRMR was 0.00022. Weighted WLSMV diagnostics therefore supported scalar invariance in every system; this conclusion is distinct from the paired-JRR uncertainty calculation for latent means.

No preceding step showed meaningful deterioration: maximum absolute ΔCFI was 0.00050 for threshold constraints, 0.00028 for metric constraints, and 0.00049 for scalar constraints. Complete final-model loadings, thresholds, latent variances, factor correlations, and same-item residual correlations are provided in the file scalar_model_selected_parameters.csv in the analysis_outputs folder of Supplementary Data S1; Supplementary Table S7 summarizes their ranges.

### 3.4 Latent enjoyment declined in every education system

Full-sample latent changes with paired-JRR intervals are reported in Table 4 and Figure 2. All nine within-system estimates were negative, and every 95% confidence interval excluded zero. Estimates ranged from −0.553 Grade 4 latent SD in Georgia to −0.117 in the Republic of Korea. Each effect is standardized on its own system’s Grade 4 latent metric. The common negative direction is therefore comparable as a replicated qualitative pattern, but the numerical magnitudes are not on a common cross-system scale and were not subjected to a formal equality test.

**Table 4:** Design-based Grade 4-to-Grade 5 latent change in mathematics enjoyment.

Education system	*N*	Zones	Δ	SEJRR	95% CI	Replicates
Georgia	4,451	79	−0.553	0.040	[−0.631, −0.475]	158/158
Italy	4,578	76	−0.250	0.023	[−0.294, −0.206]	152/152
Jordan	6,120	115	−0.146	0.054	[−0.253, −0.040]	230/230
Kosovo	4,708	86	−0.371	0.032	[−0.434, −0.309]	172/172
Montenegro	4,517	125	−0.520	0.021	[−0.561, −0.478]	250/250
North Macedonia	4,816	87	−0.318	0.035	[−0.387, −0.250]	174/174
Republic of Korea	4,500	78	−0.117	0.021	[−0.158, −0.077]	156/156
Slovenia	4,975	75	−0.371	0.024	[−0.418, −0.323]	150/150
Sweden	5,314	81	−0.369	0.028	[−0.424, −0.315]	162/162

Δ is the Grade 5 latent mean expressed in that education system’s Grade 4 latent SD. SE and 95% CI use the complete paired-JRR procedure. The system-specific metrics do not provide a formal cross-country test of equal change magnitudes.

**Figure 2:**
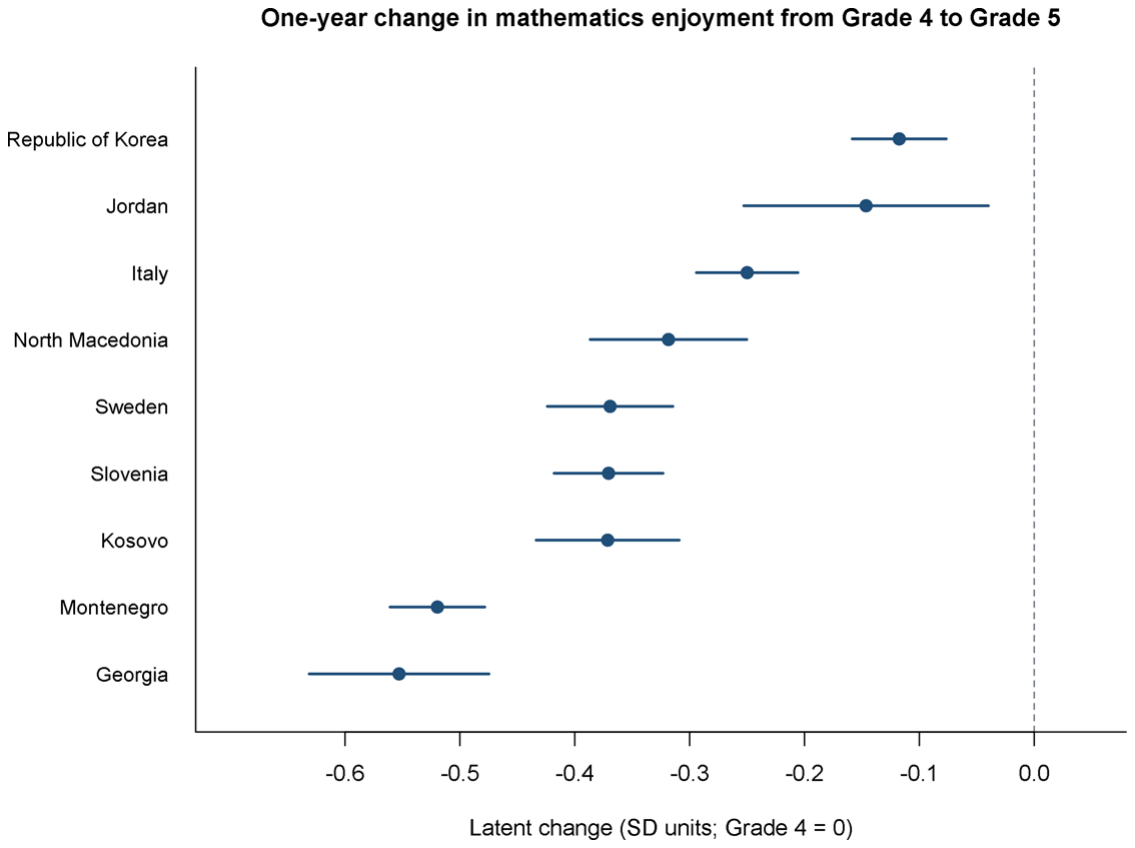
One-year change in mathematics enjoyment from Grade 4 to Grade 5 by education system. Points are scalar-model changes expressed in each system’s own Grade 4 latent standard deviation; horizontal lines are 95% paired-jackknife repeated-replication confidence intervals. The display summarizes within-system changes and is not a test of equality across systems.

### 3.5 Robustness to response definition, weighting, and follow-up selection

Every education system retained a negative latent change under all four response and weighting definitions. Across all alternative specifications, the largest absolute displacement from the weighted all-available estimate was 0.048 *SD* (Supplementary Figure S1; Supplementary Table S9). Neither the restriction to complete item response nor the removal of the sampling weights therefore accounted for the observed pattern of decrements.

Complete-covariate sample sizes for the response models ranged from 2,805 to 4,567. Across the nine systems, pooling the five plausible-value-specific models changed the baseline-enjoyment coefficient by no more than 0.0027 log-odds and its SE by less than 0.9% relative to using the mean of the five plausible values. The pooled baseline-enjoyment OR ranged from 0.878 to 1.148; only the Republic of Korea [OR = 1.12, 95% CI (1.02, 1.22)] and North Macedonia [OR = 1.15, 95% CI (1.03, 1.28)] excluded 1.00. For the achievement covariate, pooled coefficients differed by at most 0.0125 log-odds, while SE changes ranged from −3.1% to +11.9%, showing that the mean-PV sensitivity model did not systematically attenuate estimates or uniformly narrow intervals. These diagnostics reduce, but do not eliminate, concern about observed response selection.

## 4 Discussion

### 4.1 Measurement comparability preceded developmental interpretation

The present study evaluated whether mathematics enjoyment could be interpreted as the same latent construct across the transition from Grade 4 to Grade 5 before any estimate of change was interpreted. The seven content-matched positive indicators supported scalar invariance in every participating education system. The decrements in fit associated with the scalar constraints were not only smaller than the conventional benchmarks; they were of negligible magnitude and were replicated across nine independently estimated panels. The positive core can therefore be considered adequate for within-system comparison of latent means across the Grade 4 to Grade 5 interval; comparability over longer developmental spans was not evaluated.

The analysis establishes longitudinal invariance separately within each education system; it does not establish cross-country measurement equivalence. Thus, each 2024 mean may be interpreted only relative to that system’s own 2023 factor metric. Figure 2 displays system-specific standardized changes to show the replicated direction, not to rank systems or test equality of effect magnitudes. Lack of cross-country invariance testing is therefore a boundary on interpretation, not a contradictory claim that cross-country equivalence was demonstrated.

The findings further indicate that invariance should not be inferred from the recurrence of item labels. The two administrations differed in the wording composition of the broader TIMSS scale, and a composite formed from nine items in 2023 and seven items in 2024 would confound occasion with both item set and wording format. Restricting the longitudinal analysis to a content-matched core and testing ordinal invariance explicitly establishes a defensible correspondence between occasions.

### 4.2 Local dependence of negatively worded items

The two negatively worded items shared substantial residual variance in every system after conditioning on the common factor, and freeing one covariance markedly improved the nine-item model. This establishes local dependence under the specified model. It does not identify its source: negative keying, shared avoidance/boredom content, or both may contribute.

These results do not imply that the reverse-worded items carry no substantive information. Wishing not to study mathematics and finding mathematics boring may share content related to avoidance or boredom that is narrower than general enjoyment. That interpretation is nonetheless incompatible with treating the two items as conditionally independent reflections of a single unidimensional trait. Subsequent scale development should distinguish three design objectives that the present item set conflates: the measurement of positive enjoyment, the measurement of aversive or boredom-related experience, and the detection of response sets. Combining these objectives in a single total score without an explicit method or content model compromises the interpretability of the resulting construct.

The implication for large-scale assessment is narrower than a general preference for positive-only scales. Longitudinal analyses should match item content across occasions and test the resulting measurement model. When mixed-key items are retained, their local dependence and possible method/content structure should be modeled rather than assumed away by reverse scoring. The present evidence concerns this specific TIMSS item set and does not show that positive-only scales generally control response styles better.

### 4.3 A common negative direction was observed across systems

A negative within-system latent change was obtained in all nine education systems. This replicated direction is consistent with prior evidence of declining intrinsic motivation and task value during childhood (Gottfried et al., 2007; Wigfield et al., 1997; Hawrot et al., 2026). The reported range is descriptive: because each estimate uses a system-specific metric and cross-country scalar invariance was not tested, the analysis does not establish that developmental effects differ in magnitude across systems.

The apparent variation in separately standardized estimates may motivate future comparative work, but it cannot by itself establish unequal developmental effects or identify their causes. Formal comparison would require an aligned cross-country measurement model and an explicit equality test. The study followed a single cohort over one school year without random assignment of educational experiences. Curriculum, grade-transition structure, instructional climate, assessment timing, cohort composition, and post-pandemic conditions are therefore hypotheses for future research, not explanations established by these descriptive within-system changes.

### 4.4 Precision varied across systems

Re-estimating the scalar model under every paired replicate made uncertainty in the latent change estimator consistent with the supplied sampling design. JRR standard errors ranged from 0.021 to 0.054 and were not a monotone function of record count. Precision may vary with clustering, weight dispersion, missingness, item distributions, and measurement strength. Because we did not compare JRR and model-based variance components, we do not attribute the observed differences to a particular design effect.

The completeness of the replication is itself a methodological strength. All 1,604 Grade 4-to-Grade 5 replicate models converged and were admissible, so no variance estimate depended on the undocumented exclusion of failed replicates. The procedure demonstrates that nonlinear latent variable estimands can be brought within the design-based framework of TIMSS by treating the entire estimator, rather than a manifest summary of it, as the statistic recomputed under replication.

### 4.5 Strengths and limitations

The strengths of the study include student-level longitudinal linkage, coverage of nine education systems, an identification strategy appropriate to ordered categorical indicators, a content-matched item core, direct wording diagnostics, complete paired-JRR uncertainty estimation, and multiple response and weighting sensitivity analyses. Construct definition, measurement, development, and sampling were addressed within one analytic framework rather than sequentially and in isolation.

Several limitations qualify the findings. First, two occasions support a one-year change estimate, not a growth trajectory. Second, scalar invariance was established within systems, not across countries or languages; numerical differences among separately standardized changes were not formally tested. The study focused on longitudinal measurement comparability and change within each education system. Formal comparisons of change magnitudes across systems would require establishing a common cross-country measurement metric and testing equality of change parameters, which were beyond the present analytical scope. Third, the baseline diagnostic cannot distinguish negative-wording effects from shared avoidance/boredom content. Fourth, weighted WLSMV fit indices did not receive a separate cluster correction; paired-JRR intervals provide design-based uncertainty for latent change conditional on the scalar model, not design-adjusted model-selection evidence. Fifth, TOTWGT addresses sampling and unit participation but not focal item missingness, and the response restrictions and pooled-PV selection models cannot remove bias from unmeasured nonresponse. Finally, the analyses were not preregistered.

### 4.6 Implications for construct measurement and developmental inference

Three recommendations follow. First, developmental studies should define a content-matched longitudinal core and test ordinal scalar invariance before interpreting change. Second, mixed-key scales should be evaluated for local dependence and possible wording/content structure; reverse scoring is a coding operation, not evidence of conditional independence. Third, latent estimators should be carried through the supplied replicate-weight procedure when design-based uncertainty is required. These recommendations concern transparent measurement and inference, not a general claim that positive-only scales are psychometrically superior.

For users of TIMSS data, the seven positive items provide a defensible basis for the study of within-country one-year change in mathematics enjoyment in the 2023 longitudinal sample. Investigators concerned with the full baseline scale should model the two reverse-worded items explicitly rather than treat all nine items as conditionally independent indicators. Explanatory research can build on these measurement results by examining classroom-level and school-level predictors of latent change, preferably within a preregistered multilevel design that also addresses cross-national measurement alignment.

## 5 Conclusion

The same sampled Grade 4 students were linked and reassessed in Grade 5, yet valid analysis still required that linkage be distinguished from observed item response and that comparability of measurement be tested rather than assumed. Across nine education systems, the seven positively worded mathematics-enjoyment items supported scalar invariance over one school year, whereas the two reverse-worded baseline items exhibited substantial local dependence. This dependence may reflect wording direction, shared aversive content, or both. After all 1,604 prescribed paired jackknife replicate models had been incorporated, latent enjoyment decreased in every system, with decrements ranging from 0.117 to 0.553 baseline standard deviations. Developmental conclusions about non-cognitive constructs acquire greater credibility when construct definition, item wording, longitudinal invariance, missing response, and complex sampling are addressed within a single measurement framework.

## Data Availability

Publicly available datasets were analyzed in this study. The TIMSS 2023 Longitudinal Grade 4 and 5 International Database, Version 1, is available through the IEA Data Repository at https://www.iea.nl/data-tools/repository (dataset DOI: https://doi.org/10.58150/iea_timss_2023_longitudinal_g5_data_edition_1).
