# Supplementary material for: Measuring change in mathematics enjoyment: longitudinal invariance, local dependence of negatively worded items, and complex-sample inference
Source: Front Psychol. 2026 Oct 26;17:1971121. doi: 10.3389/fpsyg.2026.1971121 (PMC13745548; doi:10.3389/fpsyg.2026.1971121)
Supplement: Supplementary file 2 [file Supplementary_file_1.docx]

Supplementary Material

# Measuring Change in Mathematics Enjoyment: Longitudinal Invariance, Local Dependence of Negatively Worded Items, and Complex-Sample Inference

Georgios Sideridis and Mohammed Alghamdi

Manuscript ID: 1971121

# A. Scope and reproducibility

This supplement provides the response audit, full invariance sequence, item-level response rates, pairwise sample-size audit, baseline residual diagnostics, final-model parameter summaries, plausible-value-pooled response analysis, sensitivity results, and a reproducibility map. Country models are independent; no cross-country scalar-invariance assumption is imposed. Tables S1–S10 and Figure S1 are contained in this document. The exact machine-readable outputs, R scripts, and generated lavaan syntax are provided in the separate archive 1971121_Supplementary_Data_S1.zip (Supplementary Data S1). The Word supplement alone does not contain those files. Extract the ZIP archive and open README.md and FILE_MANIFEST.csv for the complete file inventory; paths in the reproducibility map are relative to its Supplementary_Data_S1 folder.

Supplementary Data S1 does not redistribute the TIMSS microdata. The public-use Grade 4 and 5 release can be obtained under IEA’s terms at https://doi.org/10.58150/iea_timss_2023_longitudinal_g5_data_edition_1.

# B. Item mapping and questionnaire revision

The two additional 2023 items were ASBM02B (“wish [I] did not have to study mathematics”) and ASBM02C (“mathematics is boring”). The TIMSS technical report describes the general committee-led 2024 questionnaire revision process but states that its summary does not provide a list of item-by-item edits. No published rationale specific to omission of these two items was located. Table S10 maps the seven content-matched positive items across occasions.

# Table S1. Complete longitudinal invariance sequence

| **System** | **Model** | **N** | **χ²** | **df** | **CFI** | **TLI** | **RMSEA [90% CI]** | **SRMR** | **ΔCFI** | **ΔRMSEA** | **ΔSRMR** |
| --- | --- | --- | --- | --- | --- | --- | --- | --- | --- | --- | --- |
| Georgia | Configural | 4,451 | 372.24 | 69 | .99538 | .99390 | .0314 [.0283, .0346] | .03238 | — | — | — |
| Georgia | Thresholds | 4,451 | 390.69 | 76 | .99520 | .99425 | .0305 [.0275, .0335] | .03238 | -.000175 | -.000922 | .000000 |
| Georgia | Metric | 4,451 | 400.57 | 82 | .99514 | .99461 | .0295 [.0267, .0325] | .03227 | -.000059 | -.000957 | -.000104 |
| Georgia | Scalar | 4,451 | 403.87 | 88 | .99518 | .99502 | .0284 [.0256, .0312] | .03232 | .000041 | -.001146 | .000049 |
| Italy | Configural | 4,578 | 701.52 | 69 | .99640 | .99525 | .0448 [.0418, .0478] | .02293 | — | — | — |
| Italy | Thresholds | 4,578 | 727.99 | 76 | .99628 | .99555 | .0433 [.0405, .0462] | .02293 | -.000111 | -.001459 | .000000 |
| Italy | Metric | 4,578 | 742.72 | 82 | .99623 | .99582 | .0420 [.0392, .0448] | .02293 | -.000050 | -.001336 | .000009 |
| Italy | Scalar | 4,578 | 814.17 | 88 | .99586 | .99572 | .0425 [.0398, .0452] | .02313 | -.000373 | .000503 | .000194 |
| Jordan | Configural | 6,120 | 334.18 | 69 | .99655 | .99545 | .0251 [.0224, .0278] | .02417 | — | — | — |
| Jordan | Thresholds | 6,120 | 350.11 | 76 | .99643 | .99573 | .0243 [.0217, .0269] | .02417 | -.000116 | -.000784 | .000000 |
| Jordan | Metric | 6,120 | 356.00 | 82 | .99643 | .99604 | .0234 [.0209, .0259] | .02416 | .000001 | -.000910 | .000005 |
| Jordan | Scalar | 6,120 | 379.72 | 88 | .99620 | .99607 | .0233 [.0209, .0257] | .02394 | -.000231 | -.000093 | -.000223 |
| Kosovo | Configural | 4,708 | 401.66 | 69 | .99428 | .99246 | .0320 [.0290, .0351] | .03370 | — | — | — |
| Kosovo | Thresholds | 4,708 | 437.74 | 76 | .99378 | .99256 | .0318 [.0289, .0347] | .03370 | -.000500 | -.000204 | .000000 |
| Kosovo | Metric | 4,708 | 445.71 | 82 | .99375 | .99306 | .0307 [.0279, .0335] | .03372 | -.000034 | -.001102 | .000021 |
| Kosovo | Scalar | 4,708 | 427.71 | 88 | .99416 | .99396 | .0286 [.0260, .0314] | .03363 | .000413 | -.002059 | -.000088 |
| Montenegro | Configural | 4,517 | 612.60 | 69 | .99647 | .99535 | .0418 [.0388, .0448] | .02203 | — | — | — |
| Montenegro | Thresholds | 4,517 | 667.42 | 76 | .99616 | .99540 | .0415 [.0386, .0444] | .02203 | -.000311 | -.000256 | .000000 |
| Montenegro | Metric | 4,517 | 701.29 | 82 | .99598 | .99554 | .0409 [.0381, .0437] | .02202 | -.000181 | -.000617 | -.000012 |
| Montenegro | Scalar | 4,517 | 712.98 | 88 | .99594 | .99580 | .0397 [.0370, .0424] | .02220 | -.000037 | -.001238 | .000179 |
| North Macedonia | Configural | 4,816 | 369.01 | 69 | .99705 | .99611 | .0301 [.0271, .0331] | .02238 | — | — | — |
| North Macedonia | Thresholds | 4,816 | 382.75 | 76 | .99699 | .99639 | .0290 [.0261, .0319] | .02238 | -.000066 | -.001097 | .000000 |
| North Macedonia | Metric | 4,816 | 411.37 | 82 | .99676 | .99641 | .0289 [.0261, .0317] | .02233 | -.000222 | -.000070 | -.000044 |
| North Macedonia | Scalar | 4,816 | 407.97 | 88 | .99686 | .99675 | .0275 [.0248, .0302] | .02219 | .000092 | -.001403 | -.000137 |
| Republic of Korea | Configural | 4,500 | 876.18 | 69 | .99657 | .99548 | .0510 [.0480, .0540] | .01841 | — | — | — |
| Republic of Korea | Thresholds | 4,500 | 911.80 | 76 | .99645 | .99575 | .0494 [.0466, .0523] | .01841 | -.000122 | -.001551 | .000000 |
| Republic of Korea | Metric | 4,500 | 982.89 | 82 | .99618 | .99576 | .0494 [.0467, .0522] | .01849 | -.000276 | -.000025 | .000082 |
| Republic of Korea | Scalar | 4,500 | 1010.34 | 88 | .99608 | .99595 | .0483 [.0456, .0510] | .01852 | -.000091 | -.001150 | .000030 |
| Slovenia | Configural | 4,975 | 1146.50 | 69 | .99490 | .99328 | .0560 [.0532, .0589] | .02295 | — | — | — |
| Slovenia | Thresholds | 4,975 | 1179.97 | 76 | .99478 | .99374 | .0540 [.0513, .0568] | .02294 | -.000125 | -.001991 | .000000 |
| Slovenia | Metric | 4,975 | 1201.60 | 82 | .99470 | .99412 | .0524 [.0498, .0550] | .02293 | -.000074 | -.001648 | -.000011 |
| Slovenia | Scalar | 4,975 | 1310.21 | 88 | .99422 | .99402 | .0528 [.0503, .0554] | .02301 | -.000486 | .000449 | .000080 |
| Sweden | Configural | 5,314 | 883.78 | 69 | .99628 | .99509 | .0471 [.0444, .0499] | .02058 | — | — | — |
| Sweden | Thresholds | 5,314 | 906.91 | 76 | .99620 | .99545 | .0454 [.0427, .0480] | .02058 | -.000074 | -.001781 | .000000 |
| Sweden | Metric | 5,314 | 954.89 | 82 | .99601 | .99557 | .0448 [.0422, .0473] | .02067 | -.000192 | -.000601 | .000089 |
| Sweden | Scalar | 5,314 | 993.90 | 88 | .99586 | .99572 | .0440 [.0416, .0465] | .02067 | -.000151 | -.000744 | .000002 |

Note. Δ compares a model with the immediately preceding invariance step. Weighted WLSMV diagnostics do not include a separate school-cluster correction. Model-specific scaling corrections mean that scaled WLSMV χ² need not increase monotonically across nested models; the metric-to-scalar decreases for North Macedonia and Kosovo do not by themselves indicate a nesting error.

# Table S2. Response-flow audit

| **System** | **Linked N** | **Analytic N** | **Baseline only** | **Follow-up only** | **Both waves** | **Neither** | **Both-wave %** |
| --- | --- | --- | --- | --- | --- | --- | --- |
| Georgia | 4,491 | 4,451 | 651 | 237 | 3,563 | 40 | 79.3 |
| Italy | 4,610 | 4,578 | 320 | 351 | 3,907 | 32 | 84.8 |
| Jordan | 6,172 | 6,120 | 976 | 280 | 4,864 | 52 | 78.8 |
| Kosovo | 4,747 | 4,708 | 471 | 246 | 3,991 | 39 | 84.1 |
| Montenegro | 4,559 | 4,517 | 351 | 279 | 3,887 | 42 | 85.3 |
| North Macedonia | 4,866 | 4,816 | 535 | 345 | 3,936 | 50 | 80.9 |
| Republic of Korea | 4,507 | 4,500 | 806 | 159 | 3,535 | 7 | 78.4 |
| Slovenia | 5,015 | 4,975 | 635 | 374 | 3,966 | 40 | 79.1 |
| Sweden | 5,358 | 5,314 | 678 | 361 | 4,275 | 44 | 79.8 |

# Table S3. Item-level response rates

| **System** | **Wave** | **Item** | **Observed N** | **Response %** | **Weighted response %** |
| --- | --- | --- | --- | --- | --- |
| Georgia | 2023 | Enjoy learning mathematics | 4,160 | 92.6 | 92.8 |
| Georgia | 2023 | Learn interesting things | 3,948 | 87.9 | 87.9 |
| Georgia | 2023 | Like mathematics | 3,982 | 88.7 | 88.9 |
| Georgia | 2023 | Like schoolwork involving numbers | 3,964 | 88.3 | 88.2 |
| Georgia | 2023 | Like mathematics problems | 4,007 | 89.2 | 89.3 |
| Georgia | 2023 | Look forward to mathematics lessons | 3,973 | 88.5 | 88.7 |
| Georgia | 2023 | Mathematics is a favorite subject | 3,980 | 88.6 | 88.6 |
| Georgia | 2024 | Enjoy learning mathematics | 3,739 | 83.3 | 83.8 |
| Georgia | 2024 | Learn interesting things | 3,665 | 81.6 | 82.0 |
| Georgia | 2024 | Like mathematics | 3,657 | 81.4 | 81.7 |
| Georgia | 2024 | Like schoolwork involving numbers | 3,633 | 80.9 | 81.2 |
| Georgia | 2024 | Like mathematics problems | 3,656 | 81.4 | 81.8 |
| Georgia | 2024 | Look forward to mathematics lessons | 3,647 | 81.2 | 81.7 |
| Georgia | 2024 | Mathematics is a favorite subject | 3,657 | 81.4 | 81.7 |
| Italy | 2023 | Enjoy learning mathematics | 4,213 | 91.4 | 91.5 |
| Italy | 2023 | Learn interesting things | 4,175 | 90.6 | 90.6 |
| Italy | 2023 | Like mathematics | 4,188 | 90.8 | 91.0 |
| Italy | 2023 | Like schoolwork involving numbers | 4,164 | 90.3 | 90.4 |
| Italy | 2023 | Like mathematics problems | 4,182 | 90.7 | 90.8 |
| Italy | 2023 | Look forward to mathematics lessons | 4,164 | 90.3 | 90.4 |
| Italy | 2023 | Mathematics is a favorite subject | 4,178 | 90.6 | 90.7 |
| Italy | 2024 | Enjoy learning mathematics | 4,251 | 92.2 | 92.6 |
| Italy | 2024 | Learn interesting things | 4,220 | 91.5 | 91.9 |
| Italy | 2024 | Like mathematics | 4,227 | 91.7 | 92.1 |
| Italy | 2024 | Like schoolwork involving numbers | 4,223 | 91.6 | 92.1 |
| Italy | 2024 | Like mathematics problems | 4,234 | 91.8 | 92.2 |
| Italy | 2024 | Look forward to mathematics lessons | 4,226 | 91.7 | 92.1 |
| Italy | 2024 | Mathematics is a favorite subject | 4,234 | 91.8 | 92.3 |
| Jordan | 2023 | Enjoy learning mathematics | 5,640 | 91.4 | 92.3 |
| Jordan | 2023 | Learn interesting things | 5,485 | 88.9 | 90.1 |
| Jordan | 2023 | Like mathematics | 5,491 | 89.0 | 89.9 |
| Jordan | 2023 | Like schoolwork involving numbers | 5,450 | 88.3 | 89.4 |
| Jordan | 2023 | Like mathematics problems | 5,397 | 87.4 | 88.5 |
| Jordan | 2023 | Look forward to mathematics lessons | 5,327 | 86.3 | 87.7 |
| Jordan | 2023 | Mathematics is a favorite subject | 5,360 | 86.8 | 87.9 |
| Jordan | 2024 | Enjoy learning mathematics | 4,985 | 80.8 | 80.2 |
| Jordan | 2024 | Learn interesting things | 4,908 | 79.5 | 79.1 |
| Jordan | 2024 | Like mathematics | 4,944 | 80.1 | 79.3 |
| Jordan | 2024 | Like schoolwork involving numbers | 4,927 | 79.8 | 79.1 |
| Jordan | 2024 | Like mathematics problems | 4,895 | 79.3 | 78.9 |
| Jordan | 2024 | Look forward to mathematics lessons | 4,846 | 78.5 | 78.2 |
| Jordan | 2024 | Mathematics is a favorite subject | 4,853 | 78.6 | 78.2 |
| Kosovo | 2023 | Enjoy learning mathematics | 4,405 | 92.8 | 92.3 |
| Kosovo | 2023 | Learn interesting things | 4,169 | 87.8 | 87.0 |
| Kosovo | 2023 | Like mathematics | 4,213 | 88.8 | 88.0 |
| Kosovo | 2023 | Like schoolwork involving numbers | 4,204 | 88.6 | 87.4 |
| Kosovo | 2023 | Like mathematics problems | 4,218 | 88.9 | 87.9 |
| Kosovo | 2023 | Look forward to mathematics lessons | 4,132 | 87.0 | 86.0 |
| Kosovo | 2023 | Mathematics is a favorite subject | 4,188 | 88.2 | 87.3 |
| Kosovo | 2024 | Enjoy learning mathematics | 4,189 | 88.2 | 87.9 |
| Kosovo | 2024 | Learn interesting things | 4,098 | 86.3 | 85.8 |
| Kosovo | 2024 | Like mathematics | 4,083 | 86.0 | 85.5 |
| Kosovo | 2024 | Like schoolwork involving numbers | 4,078 | 85.9 | 85.1 |
| Kosovo | 2024 | Like mathematics problems | 4,072 | 85.8 | 85.1 |
| Kosovo | 2024 | Look forward to mathematics lessons | 4,011 | 84.5 | 83.9 |
| Kosovo | 2024 | Mathematics is a favorite subject | 4,057 | 85.5 | 84.6 |
| Montenegro | 2023 | Enjoy learning mathematics | 4,155 | 91.1 | 90.9 |
| Montenegro | 2023 | Learn interesting things | 3,966 | 87.0 | 86.7 |
| Montenegro | 2023 | Like mathematics | 4,040 | 88.6 | 88.5 |
| Montenegro | 2023 | Like schoolwork involving numbers | 3,973 | 87.1 | 86.9 |
| Montenegro | 2023 | Like mathematics problems | 4,012 | 88.0 | 87.7 |
| Montenegro | 2023 | Look forward to mathematics lessons | 3,990 | 87.5 | 87.3 |
| Montenegro | 2023 | Mathematics is a favorite subject | 3,992 | 87.6 | 87.3 |
| Montenegro | 2024 | Enjoy learning mathematics | 4,075 | 89.4 | 89.4 |
| Montenegro | 2024 | Learn interesting things | 4,012 | 88.0 | 88.1 |
| Montenegro | 2024 | Like mathematics | 4,003 | 87.8 | 87.9 |
| Montenegro | 2024 | Like schoolwork involving numbers | 3,949 | 86.6 | 86.8 |
| Montenegro | 2024 | Like mathematics problems | 3,986 | 87.4 | 87.5 |
| Montenegro | 2024 | Look forward to mathematics lessons | 3,975 | 87.2 | 87.4 |
| Montenegro | 2024 | Mathematics is a favorite subject | 3,992 | 87.6 | 87.7 |
| North Macedonia | 2023 | Enjoy learning mathematics | 4,350 | 89.4 | 89.8 |
| North Macedonia | 2023 | Learn interesting things | 3,981 | 81.8 | 82.5 |
| North Macedonia | 2023 | Like mathematics | 4,043 | 83.1 | 83.5 |
| North Macedonia | 2023 | Like schoolwork involving numbers | 4,016 | 82.5 | 82.8 |
| North Macedonia | 2023 | Like mathematics problems | 3,994 | 82.1 | 82.2 |
| North Macedonia | 2023 | Look forward to mathematics lessons | 3,974 | 81.7 | 81.9 |
| North Macedonia | 2023 | Mathematics is a favorite subject | 3,995 | 82.1 | 82.3 |
| North Macedonia | 2024 | Enjoy learning mathematics | 4,147 | 85.2 | 85.3 |
| North Macedonia | 2024 | Learn interesting things | 3,992 | 82.0 | 81.9 |
| North Macedonia | 2024 | Like mathematics | 3,999 | 82.2 | 81.9 |
| North Macedonia | 2024 | Like schoolwork involving numbers | 3,973 | 81.6 | 81.3 |
| North Macedonia | 2024 | Like mathematics problems | 3,985 | 81.9 | 81.6 |
| North Macedonia | 2024 | Look forward to mathematics lessons | 3,928 | 80.7 | 80.4 |
| North Macedonia | 2024 | Mathematics is a favorite subject | 3,939 | 80.9 | 80.5 |
| Republic of Korea | 2023 | Enjoy learning mathematics | 4,338 | 96.3 | 96.2 |
| Republic of Korea | 2023 | Learn interesting things | 4,321 | 95.9 | 95.8 |
| Republic of Korea | 2023 | Like mathematics | 4,322 | 95.9 | 95.8 |
| Republic of Korea | 2023 | Like schoolwork involving numbers | 4,324 | 95.9 | 95.9 |
| Republic of Korea | 2023 | Like mathematics problems | 4,319 | 95.8 | 95.7 |
| Republic of Korea | 2023 | Look forward to mathematics lessons | 4,302 | 95.5 | 95.4 |
| Republic of Korea | 2023 | Mathematics is a favorite subject | 4,325 | 96.0 | 95.9 |
| Republic of Korea | 2024 | Enjoy learning mathematics | 3,693 | 81.9 | 84.4 |
| Republic of Korea | 2024 | Learn interesting things | 3,685 | 81.8 | 84.2 |
| Republic of Korea | 2024 | Like mathematics | 3,684 | 81.7 | 84.2 |
| Republic of Korea | 2024 | Like schoolwork involving numbers | 3,686 | 81.8 | 84.2 |
| Republic of Korea | 2024 | Like mathematics problems | 3,679 | 81.6 | 84.1 |
| Republic of Korea | 2024 | Look forward to mathematics lessons | 3,684 | 81.7 | 84.2 |
| Republic of Korea | 2024 | Mathematics is a favorite subject | 3,692 | 81.9 | 84.4 |
| Slovenia | 2023 | Enjoy learning mathematics | 4,579 | 91.3 | 91.0 |
| Slovenia | 2023 | Learn interesting things | 4,549 | 90.7 | 90.4 |
| Slovenia | 2023 | Like mathematics | 4,515 | 90.0 | 89.7 |
| Slovenia | 2023 | Like schoolwork involving numbers | 4,531 | 90.3 | 89.9 |
| Slovenia | 2023 | Like mathematics problems | 4,548 | 90.7 | 90.3 |
| Slovenia | 2023 | Look forward to mathematics lessons | 4,533 | 90.4 | 90.1 |
| Slovenia | 2023 | Mathematics is a favorite subject | 4,532 | 90.4 | 90.1 |
| Slovenia | 2024 | Enjoy learning mathematics | 4,333 | 86.4 | 87.0 |
| Slovenia | 2024 | Learn interesting things | 4,324 | 86.2 | 86.8 |
| Slovenia | 2024 | Like mathematics | 4,303 | 85.8 | 86.4 |
| Slovenia | 2024 | Like schoolwork involving numbers | 4,316 | 86.1 | 86.7 |
| Slovenia | 2024 | Like mathematics problems | 4,324 | 86.2 | 86.8 |
| Slovenia | 2024 | Look forward to mathematics lessons | 4,305 | 85.8 | 86.4 |
| Slovenia | 2024 | Mathematics is a favorite subject | 4,313 | 86.0 | 86.6 |
| Sweden | 2023 | Enjoy learning mathematics | 4,942 | 92.2 | 92.6 |
| Sweden | 2023 | Learn interesting things | 4,899 | 91.4 | 91.8 |
| Sweden | 2023 | Like mathematics | 4,873 | 90.9 | 91.2 |
| Sweden | 2023 | Like schoolwork involving numbers | 4,905 | 91.5 | 91.9 |
| Sweden | 2023 | Like mathematics problems | 4,913 | 91.7 | 92.1 |
| Sweden | 2023 | Look forward to mathematics lessons | 4,893 | 91.3 | 91.8 |
| Sweden | 2023 | Mathematics is a favorite subject | 4,915 | 91.7 | 92.1 |
| Sweden | 2024 | Enjoy learning mathematics | 4,626 | 86.3 | 86.8 |
| Sweden | 2024 | Learn interesting things | 4,607 | 86.0 | 86.4 |
| Sweden | 2024 | Like mathematics | 4,597 | 85.8 | 86.2 |
| Sweden | 2024 | Like schoolwork involving numbers | 4,596 | 85.8 | 86.3 |
| Sweden | 2024 | Like mathematics problems | 4,608 | 86.0 | 86.4 |
| Sweden | 2024 | Look forward to mathematics lessons | 4,598 | 85.8 | 86.2 |
| Sweden | 2024 | Mathematics is a favorite subject | 4,605 | 85.9 | 86.4 |

# Table S4. Pairwise sample-size ranges

| **System** | **Minimum N** | **Minimum pair** | **Maximum N** | **Maximum pair** |
| --- | --- | --- | --- | --- |
| Georgia | 3,242 | m23_learn_interesting / m24_like_number_work | 3,981 | m23_enjoy_learning / m23_like_problems |
| Italy | 3,819 | m23_like_number_work / m24_learn_interesting | 4,230 | m24_enjoy_learning / m24_like_problems |
| Jordan | 4,236 | m23_look_forward / m24_look_forward | 5,390 | m23_enjoy_learning / m23_learn_interesting |
| Kosovo | 3,562 | m23_look_forward / m24_look_forward | 4,192 | m23_enjoy_learning / m23_like_problems |
| Montenegro | 3,490 | m23_learn_interesting / m24_like_number_work | 3,997 | m23_enjoy_learning / m23_like_mathematics |
| North Macedonia | 3,332 | m23_learn_interesting / m24_look_forward | 3,999 | m23_enjoy_learning / m23_like_mathematics |
| Republic of Korea | 3,489 | m23_look_forward / m24_like_problems | 4,322 | m23_enjoy_learning / m23_favorite_subject |
| Slovenia | 3,861 | m23_like_mathematics / m24_like_mathematics | 4,537 | m23_enjoy_learning / m23_like_problems |
| Sweden | 4,180 | m23_like_mathematics / m24_like_number_work | 4,908 | m23_enjoy_learning / m23_favorite_subject |

# Table S5. Baseline model fit with RMSEA intervals

| **System** | **Model** | **N** | **χ²** | **df** | **CFI** | **TLI** | **RMSEA [90% CI]** | **SRMR** | **Max \|resid\|** | **Residual pair** |
| --- | --- | --- | --- | --- | --- | --- | --- | --- | --- | --- |
| Georgia | Seven positive | 4,214 | 233.47 | 14 | .9922 | .9883 | .0610 [.0543, .0680] | .0296 | .0651 | p_like_number_work / p_favorite_subject |
| Georgia | Nine-item one factor | 4,229 | 2299.80 | 27 | .9313 | .9083 | .1411 [.1362, .1460] | .0934 | .3065 | n_wish_no_math / n_math_boring |
| Georgia | Nine-item + residual covariance | 4,229 | 230.73 | 26 | .9938 | .9914 | .0432 [.0381, .0484] | .0253 | .0637 | p_like_number_work / p_favorite_subject |
| Italy | Seven positive | 4,227 | 410.53 | 14 | .9960 | .9940 | .0819 [.0751, .0888] | .0248 | .0676 | p_learn_interesting / p_like_number_work |
| Italy | Nine-item one factor | 4,231 | 1363.33 | 27 | .9874 | .9832 | .1082 [.1033, .1131] | .0381 | .1553 | n_wish_no_math / n_math_boring |
| Italy | Nine-item + residual covariance | 4,231 | 547.27 | 26 | .9951 | .9932 | .0688 [.0639, .0739] | .0231 | .0707 | p_learn_interesting / p_like_number_work |
| Jordan | Seven positive | 5,840 | 184.63 | 14 | .9966 | .9950 | .0457 [.0399, .0517] | .0168 | .0453 | p_learn_interesting / p_favorite_subject |
| Jordan | Nine-item one factor | 5,877 | 5449.52 | 27 | .9029 | .8705 | .1849 [.1808, .1890] | .1255 | .5561 | n_wish_no_math / n_math_boring |
| Jordan | Nine-item + residual covariance | 5,877 | 204.30 | 26 | .9968 | .9956 | .0342 [.0299, .0386] | .0211 | .0627 | p_learn_interesting / n_math_boring |
| Kosovo | Seven positive | 4,462 | 95.46 | 14 | .9949 | .9924 | .0361 [.0295, .0431] | .0215 | .0559 | p_like_problems / p_look_forward |
| Kosovo | Nine-item one factor | 4,478 | 1015.85 | 27 | .9470 | .9294 | .0904 [.0857, .0953] | .0791 | .4285 | n_wish_no_math / n_math_boring |
| Kosovo | Nine-item + residual covariance | 4,478 | 150.88 | 26 | .9933 | .9907 | .0328 [.0278, .0379] | .0304 | .0882 | p_look_forward / n_math_boring |
| Montenegro | Seven positive | 4,238 | 274.27 | 14 | .9959 | .9938 | .0662 [.0595, .0732] | .0188 | .0524 | p_learn_interesting / p_like_number_work |
| Montenegro | Nine-item one factor | 4,254 | 3395.24 | 27 | .9495 | .9327 | .1713 [.1664, .1762] | .0803 | .3749 | n_wish_no_math / n_math_boring |
| Montenegro | Nine-item + residual covariance | 4,254 | 447.54 | 26 | .9937 | .9913 | .0617 [.0568, .0668] | .0249 | .0636 | p_like_problems / n_wish_no_math |
| North Macedonia | Seven positive | 4,471 | 210.13 | 14 | .9952 | .9928 | .0560 [.0494, .0628] | .0196 | .0538 | p_like_number_work / p_favorite_subject |
| North Macedonia | Nine-item one factor | 4,513 | 3259.92 | 27 | .9289 | .9051 | .1629 [.1582, .1677] | .0985 | .4795 | n_wish_no_math / n_math_boring |
| North Macedonia | Nine-item + residual covariance | 4,513 | 291.26 | 26 | .9942 | .9919 | .0476 [.0427, .0525] | .0287 | .0758 | p_look_forward / n_math_boring |
| Republic of Korea | Seven positive | 4,341 | 500.00 | 14 | .9959 | .9938 | .0894 [.0828, .0962] | .0180 | .0450 | p_like_number_work / p_like_problems |
| Republic of Korea | Nine-item one factor | 4,347 | 2137.42 | 27 | .9823 | .9764 | .1341 [.1293, .1390] | .0458 | .2261 | n_wish_no_math / n_math_boring |
| Republic of Korea | Nine-item + residual covariance | 4,347 | 640.91 | 26 | .9949 | .9929 | .0738 [.0689, .0788] | .0180 | .0486 | p_like_number_work / p_like_problems |
| Slovenia | Seven positive | 4,601 | 567.13 | 14 | .9946 | .9919 | .0927 [.0862, .0993] | .0215 | .0514 | p_learn_interesting / p_favorite_subject |
| Slovenia | Nine-item one factor | 4,608 | 2774.14 | 27 | .9739 | .9652 | .1486 [.1440, .1533] | .0567 | .2957 | n_wish_no_math / n_math_boring |
| Slovenia | Nine-item + residual covariance | 4,608 | 888.23 | 26 | .9918 | .9887 | .0848 [.0801, .0897] | .0247 | .0689 | p_like_number_work / n_math_boring |
| Sweden | Seven positive | 4,953 | 484.78 | 14 | .9956 | .9934 | .0824 [.0762, .0888] | .0213 | .0598 | p_learn_interesting / p_favorite_subject |
| Sweden | Nine-item one factor | 4,958 | 1459.93 | 27 | .9881 | .9841 | .1035 [.0990, .1080] | .0337 | .1330 | n_wish_no_math / n_math_boring |
| Sweden | Nine-item + residual covariance | 4,958 | 783.84 | 26 | .9937 | .9913 | .0767 [.0721, .0814] | .0223 | .0574 | p_learn_interesting / p_favorite_subject |

# Table S6. Three largest residual-correlation discrepancies per baseline model

| **System** | **Model** | **Rank** | **Item 1** | **Item 2** | **Residual r** |
| --- | --- | --- | --- | --- | --- |
| Georgia | Seven positive | 1 | p_like_number_work | p_favorite_subject | -.0651 |
| Georgia | Seven positive | 2 | p_learn_interesting | p_favorite_subject | -.0607 |
| Georgia | Seven positive | 3 | p_learn_interesting | p_like_number_work | .0578 |
| Georgia | Nine-item + residual covariance | 1 | p_like_number_work | p_favorite_subject | -.0637 |
| Georgia | Nine-item + residual covariance | 2 | p_learn_interesting | p_favorite_subject | -.0603 |
| Georgia | Nine-item + residual covariance | 3 | p_learn_interesting | p_like_number_work | .0599 |
| Georgia | Nine-item one factor | 1 | n_wish_no_math | n_math_boring | .3065 |
| Georgia | Nine-item one factor | 2 | p_enjoy_learning | n_wish_no_math | -.1720 |
| Georgia | Nine-item one factor | 3 | p_like_problems | n_wish_no_math | -.1657 |
| Italy | Seven positive | 1 | p_learn_interesting | p_like_number_work | .0676 |
| Italy | Seven positive | 2 | p_like_number_work | p_like_problems | .0576 |
| Italy | Seven positive | 3 | p_like_problems | p_favorite_subject | -.0409 |
| Italy | Nine-item + residual covariance | 1 | p_learn_interesting | p_like_number_work | .0707 |
| Italy | Nine-item + residual covariance | 2 | p_like_number_work | p_like_problems | .0666 |
| Italy | Nine-item + residual covariance | 3 | p_learn_interesting | p_favorite_subject | -.0375 |
| Italy | Nine-item one factor | 1 | n_wish_no_math | n_math_boring | .1553 |
| Italy | Nine-item one factor | 2 | p_learn_interesting | p_like_number_work | .0758 |
| Italy | Nine-item one factor | 3 | p_like_number_work | p_like_problems | .0717 |
| Jordan | Seven positive | 1 | p_learn_interesting | p_favorite_subject | -.0453 |
| Jordan | Seven positive | 2 | p_learn_interesting | p_like_number_work | .0335 |
| Jordan | Seven positive | 3 | p_enjoy_learning | p_learn_interesting | -.0300 |
| Jordan | Nine-item + residual covariance | 1 | p_learn_interesting | n_math_boring | -.0627 |
| Jordan | Nine-item + residual covariance | 2 | p_like_mathematics | n_math_boring | .0487 |
| Jordan | Nine-item + residual covariance | 3 | p_learn_interesting | p_favorite_subject | -.0444 |
| Jordan | Nine-item one factor | 1 | n_wish_no_math | n_math_boring | .5561 |
| Jordan | Nine-item one factor | 2 | p_like_number_work | n_wish_no_math | -.2123 |
| Jordan | Nine-item one factor | 3 | p_look_forward | n_wish_no_math | -.2006 |
| Kosovo | Seven positive | 1 | p_like_problems | p_look_forward | .0559 |
| Kosovo | Seven positive | 2 | p_like_mathematics | p_look_forward | -.0353 |
| Kosovo | Seven positive | 3 | p_enjoy_learning | p_look_forward | -.0347 |
| Kosovo | Nine-item + residual covariance | 1 | p_look_forward | n_math_boring | -.0882 |
| Kosovo | Nine-item + residual covariance | 2 | p_look_forward | n_wish_no_math | -.0772 |
| Kosovo | Nine-item + residual covariance | 3 | p_like_problems | p_look_forward | .0644 |
| Kosovo | Nine-item one factor | 1 | n_wish_no_math | n_math_boring | .4285 |
| Kosovo | Nine-item one factor | 2 | p_look_forward | n_wish_no_math | -.1519 |
| Kosovo | Nine-item one factor | 3 | p_look_forward | n_math_boring | -.1190 |
| Montenegro | Seven positive | 1 | p_learn_interesting | p_like_number_work | .0524 |
| Montenegro | Seven positive | 2 | p_learn_interesting | p_favorite_subject | -.0487 |
| Montenegro | Seven positive | 3 | p_like_number_work | p_favorite_subject | -.0354 |
| Montenegro | Nine-item + residual covariance | 1 | p_like_problems | n_wish_no_math | -.0636 |
| Montenegro | Nine-item + residual covariance | 2 | p_like_mathematics | n_math_boring | .0602 |
| Montenegro | Nine-item + residual covariance | 3 | p_learn_interesting | p_like_number_work | .0533 |
| Montenegro | Nine-item one factor | 1 | n_wish_no_math | n_math_boring | .3749 |
| Montenegro | Nine-item one factor | 2 | p_like_problems | n_wish_no_math | -.1698 |
| Montenegro | Nine-item one factor | 3 | p_like_number_work | n_wish_no_math | -.1477 |
| North Macedonia | Seven positive | 1 | p_like_number_work | p_favorite_subject | -.0538 |
| North Macedonia | Seven positive | 2 | p_learn_interesting | p_like_number_work | .0468 |
| North Macedonia | Seven positive | 3 | p_learn_interesting | p_favorite_subject | -.0409 |
| North Macedonia | Nine-item + residual covariance | 1 | p_look_forward | n_math_boring | -.0758 |
| North Macedonia | Nine-item + residual covariance | 2 | p_look_forward | n_wish_no_math | -.0719 |
| North Macedonia | Nine-item + residual covariance | 3 | p_like_mathematics | n_math_boring | .0703 |
| North Macedonia | Nine-item one factor | 1 | n_wish_no_math | n_math_boring | .4795 |
| North Macedonia | Nine-item one factor | 2 | p_look_forward | n_wish_no_math | -.1973 |
| North Macedonia | Nine-item one factor | 3 | p_look_forward | n_math_boring | -.1573 |
| Republic of Korea | Seven positive | 1 | p_like_number_work | p_like_problems | .0450 |
| Republic of Korea | Seven positive | 2 | p_look_forward | p_favorite_subject | .0376 |
| Republic of Korea | Seven positive | 3 | p_learn_interesting | p_favorite_subject | -.0320 |
| Republic of Korea | Nine-item + residual covariance | 1 | p_like_number_work | p_like_problems | .0486 |
| Republic of Korea | Nine-item + residual covariance | 2 | p_look_forward | p_favorite_subject | .0379 |
| Republic of Korea | Nine-item + residual covariance | 3 | p_learn_interesting | n_math_boring | -.0314 |
| Republic of Korea | Nine-item one factor | 1 | n_wish_no_math | n_math_boring | .2261 |
| Republic of Korea | Nine-item one factor | 2 | p_like_number_work | n_wish_no_math | -.0749 |
| Republic of Korea | Nine-item one factor | 3 | p_look_forward | n_wish_no_math | -.0724 |
| Slovenia | Seven positive | 1 | p_learn_interesting | p_favorite_subject | -.0514 |
| Slovenia | Seven positive | 2 | p_like_number_work | p_favorite_subject | -.0510 |
| Slovenia | Seven positive | 3 | p_learn_interesting | p_like_number_work | .0454 |
| Slovenia | Nine-item + residual covariance | 1 | p_like_number_work | n_math_boring | -.0689 |
| Slovenia | Nine-item + residual covariance | 2 | p_like_problems | n_math_boring | -.0554 |
| Slovenia | Nine-item + residual covariance | 3 | p_learn_interesting | p_favorite_subject | -.0534 |
| Slovenia | Nine-item one factor | 1 | n_wish_no_math | n_math_boring | .2957 |
| Slovenia | Nine-item one factor | 2 | p_like_number_work | n_wish_no_math | -.1074 |
| Slovenia | Nine-item one factor | 3 | p_like_number_work | n_math_boring | -.0885 |
| Sweden | Seven positive | 1 | p_learn_interesting | p_favorite_subject | -.0598 |
| Sweden | Seven positive | 2 | p_like_number_work | p_favorite_subject | -.0500 |
| Sweden | Seven positive | 3 | p_like_number_work | p_like_problems | .0443 |
| Sweden | Nine-item + residual covariance | 1 | p_learn_interesting | p_favorite_subject | -.0574 |
| Sweden | Nine-item + residual covariance | 2 | p_like_number_work | p_like_problems | .0541 |
| Sweden | Nine-item + residual covariance | 3 | p_like_number_work | n_math_boring | -.0482 |
| Sweden | Nine-item one factor | 1 | n_wish_no_math | n_math_boring | .1330 |
| Sweden | Nine-item one factor | 2 | p_like_number_work | n_wish_no_math | -.0663 |
| Sweden | Nine-item one factor | 3 | p_like_number_work | n_math_boring | -.0600 |

# Table S7. Scalar-model parameter summary

| **System** | **Std. loading range** | **Threshold range** | **Var Grade 4** | **Var Grade 5** | **Factor r** | **Same-item residual r range** |
| --- | --- | --- | --- | --- | --- | --- |
| Georgia | 0.737–0.943 | -5.464–-0.413 | 1.000 | 0.975 | 0.431 | 0.064–0.308 |
| Italy | 0.717–0.965 | -4.448–1.047 | 1.000 | 0.917 | 0.681 | 0.167–0.681 |
| Jordan | 0.784–0.929 | -3.563–-0.722 | 1.000 | 0.997 | 0.340 | -0.020–0.304 |
| Kosovo | 0.707–0.955 | -5.174–-0.654 | 1.000 | 0.953 | 0.544 | -0.115–0.558 |
| Montenegro | 0.799–0.954 | -4.168–-0.226 | 1.000 | 1.005 | 0.495 | -0.071–0.427 |
| North Macedonia | 0.820–0.955 | -5.408–-0.570 | 1.000 | 1.001 | 0.495 | 0.055–0.445 |
| Republic of Korea | 0.779–0.963 | -2.789–2.127 | 1.000 | 1.231 | 0.660 | 0.043–0.595 |
| Slovenia | 0.816–0.961 | -3.738–1.356 | 1.000 | 1.006 | 0.593 | 0.069–0.616 |
| Sweden | 0.785–0.963 | -3.867–1.814 | 1.000 | 1.209 | 0.662 | 0.128–0.576 |

Note. Exact loadings, all thresholds, latent variances/covariances, and all seven longitudinal residual correlations per system are in analysis_outputs/scalar_model_selected_parameters.csv within Supplementary Data S1 (594 parameter records across nine systems). The est and std.all columns provide unstandardized and standardized estimates, respectively; the reported standard errors and confidence limits in this file are model-based WLSMV results, not paired-JRR uncertainty for latent change.

# Table S8. Plausible-value-pooled response-selection coefficients

| **System** | **Complete-covariate N** | **Baseline enjoyment OR [95% CI]** | **Achievement OR [95% CI]** |
| --- | --- | --- | --- |
| Georgia | 3,548 | 1.036 [0.914, 1.173] | 0.941 [0.803, 1.103] |
| Italy | 3,713 | 1.074 [0.862, 1.337] | 1.234 [1.013, 1.502] |
| Jordan | 4,567 | 0.994 [0.912, 1.082] | 1.010 [0.870, 1.173] |
| Kosovo | 2,805 | 1.000 [0.863, 1.159] | 1.145 [0.967, 1.356] |
| Montenegro | 3,158 | 0.980 [0.854, 1.124] | 1.199 [1.020, 1.410] |
| North Macedonia | 3,562 | 1.148 [1.033, 1.276] | 1.052 [0.915, 1.209] |
| Republic of Korea | 4,229 | 1.117 [1.020, 1.223] | 0.914 [0.776, 1.077] |
| Slovenia | 3,809 | 0.989 [0.881, 1.110] | 1.019 [0.875, 1.186] |
| Sweden | 3,636 | 0.878 [0.764, 1.008] | 1.059 [0.907, 1.237] |

Note. Each model was fitted separately across all five mathematics plausible values with full paired-JRR sampling variance, then pooled using the TIMSS/Rubin rule. All coefficients, including categorical covariates, are supplied in analysis_outputs/attrition_models_pv_pooled_coefficients.csv. The corresponding plausible-value-specific results and comparison with the mean-PV sensitivity model described in Methods §2.7 are identified in the reproducibility map.

# Table S9. Latent-change sensitivity

| **System** | **Definition** | **N** | **Latent change** | **CFI** | **RMSEA** | **SRMR** |
| --- | --- | --- | --- | --- | --- | --- |
| Georgia | weighted all available | 4,451 | -.5529 | .9952 | .0284 | .0323 |
| Georgia | weighted min4 each wave | 3,362 | -.5633 | .9951 | .0310 | .0329 |
| Georgia | weighted complete7 both | 2,893 | -.5930 | .9951 | .0316 | .0335 |
| Georgia | unweighted all available | 4,451 | -.5092 | .9948 | .0348 | .0304 |
| Italy | weighted all available | 4,578 | -.2499 | .9959 | .0425 | .0231 |
| Italy | weighted min4 each wave | 3,882 | -.2537 | .9954 | .0469 | .0236 |
| Italy | weighted complete7 both | 3,612 | -.2547 | .9955 | .0479 | .0236 |
| Italy | unweighted all available | 4,578 | -.2430 | .9957 | .0477 | .0235 |
| Jordan | weighted all available | 6,120 | -.1464 | .9962 | .0233 | .0239 |
| Jordan | weighted min4 each wave | 4,433 | -.1624 | .9960 | .0260 | .0231 |
| Jordan | weighted complete7 both | 3,725 | -.1847 | .9965 | .0256 | .0234 |
| Jordan | unweighted all available | 6,120 | -.1207 | .9971 | .0281 | .0201 |
| Kosovo | weighted all available | 4,708 | -.3713 | .9942 | .0286 | .0336 |
| Kosovo | weighted min4 each wave | 3,747 | -.4014 | .9932 | .0329 | .0344 |
| Kosovo | weighted complete7 both | 3,170 | -.4191 | .9940 | .0318 | .0330 |
| Kosovo | unweighted all available | 4,708 | -.4063 | .9939 | .0351 | .0292 |
| Montenegro | weighted all available | 4,517 | -.5195 | .9959 | .0397 | .0222 |
| Montenegro | weighted min4 each wave | 3,632 | -.5262 | .9958 | .0435 | .0225 |
| Montenegro | weighted complete7 both | 3,102 | -.5383 | .9960 | .0448 | .0216 |
| Montenegro | unweighted all available | 4,517 | -.5098 | .9961 | .0413 | .0221 |
| North Macedonia | weighted all available | 4,816 | -.3184 | .9969 | .0275 | .0222 |
| North Macedonia | weighted min4 each wave | 3,469 | -.3566 | .9964 | .0320 | .0222 |
| North Macedonia | weighted complete7 both | 2,922 | -.3484 | .9964 | .0333 | .0221 |
| North Macedonia | unweighted all available | 4,816 | -.3200 | .9966 | .0313 | .0210 |
| Republic of Korea | weighted all available | 4,500 | -.1175 | .9961 | .0483 | .0185 |
| Republic of Korea | weighted min4 each wave | 3,534 | -.1307 | .9957 | .0536 | .0189 |
| Republic of Korea | weighted complete7 both | 3,391 | -.1252 | .9957 | .0543 | .0187 |
| Republic of Korea | unweighted all available | 4,500 | -.1233 | .9963 | .0539 | .0169 |
| Slovenia | weighted all available | 4,975 | -.3706 | .9942 | .0528 | .0230 |
| Slovenia | weighted min4 each wave | 3,933 | -.3588 | .9938 | .0584 | .0233 |
| Slovenia | weighted complete7 both | 3,669 | -.3593 | .9937 | .0599 | .0232 |
| Slovenia | unweighted all available | 4,975 | -.3835 | .9943 | .0579 | .0225 |
| Sweden | weighted all available | 5,314 | -.3692 | .9959 | .0440 | .0207 |
| Sweden | weighted min4 each wave | 4,236 | -.3638 | .9959 | .0463 | .0204 |
| Sweden | weighted complete7 both | 4,040 | -.3593 | .9958 | .0471 | .0208 |
| Sweden | unweighted all available | 5,314 | -.3548 | .9958 | .0517 | .0203 |

# Figure S1. Sensitivity of latent change

Four estimates are shown per system: weighted all available, weighted minimum four items per wave, weighted complete seven items, and unweighted all available. All remain below zero.

# Table S10. Item mapping across Grade 4 and Grade 5

| **Content** | **Grade 4 (2023)** | **Grade 5 (2024)** |
| --- | --- | --- |
| Enjoy learning mathematics | ASBM02A | ASBLM02A |
| Learn interesting things | ASBM02D | ASBLM02B |
| Like mathematics | ASBM02E | ASBLM02C |
| Like schoolwork involving numbers | ASBM02F | ASBLM02D |
| Like mathematics problems | ASBM02G | ASBLM02E |
| Look forward to mathematics lessons | ASBM02H | ASBLM02F |
| Mathematics is a favorite subject | ASBM02I | ASBLM02G |

# Reproducibility map

| **File** | **Purpose** |
| --- | --- |
| R/00_config.R | Paths, item maps, recodes, shared functions |
| R/01_audit_items.R | Identifier and item audit |
| R/02_fit_longitudinal_models.R | Four-step ordinal invariance sequence and final parameters |
| R/03_fit_wording_models.R | Baseline seven-/nine-item models |
| R/04_sensitivity_analysis.R | Response/weighting sensitivity |
| R/06_jrr_latent_change.R | Paired-JRR scalar-model re-estimation |
| R/13_reviewer_revision_analyses.R | Response, residual, and model-fit audits |
| R/14_pv_response_models.R | Five plausible-value-specific JRR response models |
| R/15_pool_pv_response_models.R | TIMSS/Rubin pooling and validation |
| models/*.lavaan | Generated configural, threshold, metric, and scalar syntax |
| analysis_outputs/*.csv | Machine-readable analysis results |
| analysis_outputs/scalar_model_selected_parameters.csv | Exact final scalar-model parameters: 594 records across nine systems, including estimates and standardized values |
| analysis_outputs/attrition_models_pv_specific_coefficients.csv | All coefficients from 45 plausible-value-specific models, with paired-JRR variances and standard errors (810 records) |
| analysis_outputs/attrition_models_pv_pooled_coefficients.csv | Pooled coefficients, within-JRR and between-PV variance, total variance, standard errors, and intervals (162 records) |
| analysis_outputs/attrition_models_pv_pooled_vs_average.csv | Comparison of pooled results with the mean-PV sensitivity model (162 records) |
| analysis_outputs/attrition_models_pv_specific_samples.csv | Analysis sample sizes for all 45 plausible-value-specific models |
| analysis_outputs/pv_pooling_validation.csv | Saved checks of plausible-value pooling |
| FILE_MANIFEST.csv | Complete file inventory with file sizes and SHA-256 checksums |
